# Arsenic induced oxidative stress and mitochondrial dysfunction in rat brain

**DOI:** 10.1186/2193-1801-4-S1-P20

**Published:** 2015-06-12

**Authors:** Vijay Kumar, Chandra Prakash

**Affiliations:** Maharshi Dayanand University, India

**Keywords:** Arsenic, oxidative stress, mitochondria

The present study was undertaken to reveal the effects of chronic arsenic exposure (25ppm intragastrically for 12 weeks) on mitochondrial functions and oxidative stress in male Wister rats. Chronic arsenic exposure resulted in decrease in the activities of the mitochondrial complexes. There was increased generation of ROS followed by decrease in MnSOD activity. The generation of oxidative stress was associated with increased protein oxidation and lipid peroxidation in rat brain as evident by FTIR spectra. The RT-PCR analysis of NRF 1, NRF 2 and PGC 1α revealed decrease in gene expression suggesting decreased biogenesis following chronic exposure in rat brain. Thus, the findings of the present study reveal that arsenic induced decrease in mitochondrial biogenesis may be responsible for the decreased metabolic response that may be further involved in the generation of oxidative stress and neurodegeneration in rat brain.

